# Spondylolysis and Spondylolisthesis in Athletes

**DOI:** 10.1055/s-0043-1777435

**Published:** 2024-03-21

**Authors:** Marcos Vaz de Lima, Maria Fernanda Silber Caffaro, Claudio Santili, Robert G. Watkins IV

**Affiliations:** 1 Grupo de Traumatologia do Esporte, Departamento de Ortopedia e Traumatologia, Faculdade de Ciências Médicas da Santa Casa de São Paulo, São Paulo, SP, Brasil; 2 Faculdade de Ciências Médicas da Santa Casa de São Paulo, São Paulo, SP, Brasil; 3 Departamento de Ortopedia e Traumatologia, Faculdade de Ciências Médicas da Santa Casa de São Paulo, São Paulo, SP, Brasil; 4 Marina Spine Center, Los Angeles, CA, Estados Unidos

**Keywords:** athletes, chronic pain, low back pain, spondylolysis, spondylolisthesis, sports

## Abstract

This article is an update on spondylolysis and spondylolisthesis in athletes, from diagnosis to treatment, based on our service experience and a literature review.

## Introduction

**Fig. 1:**
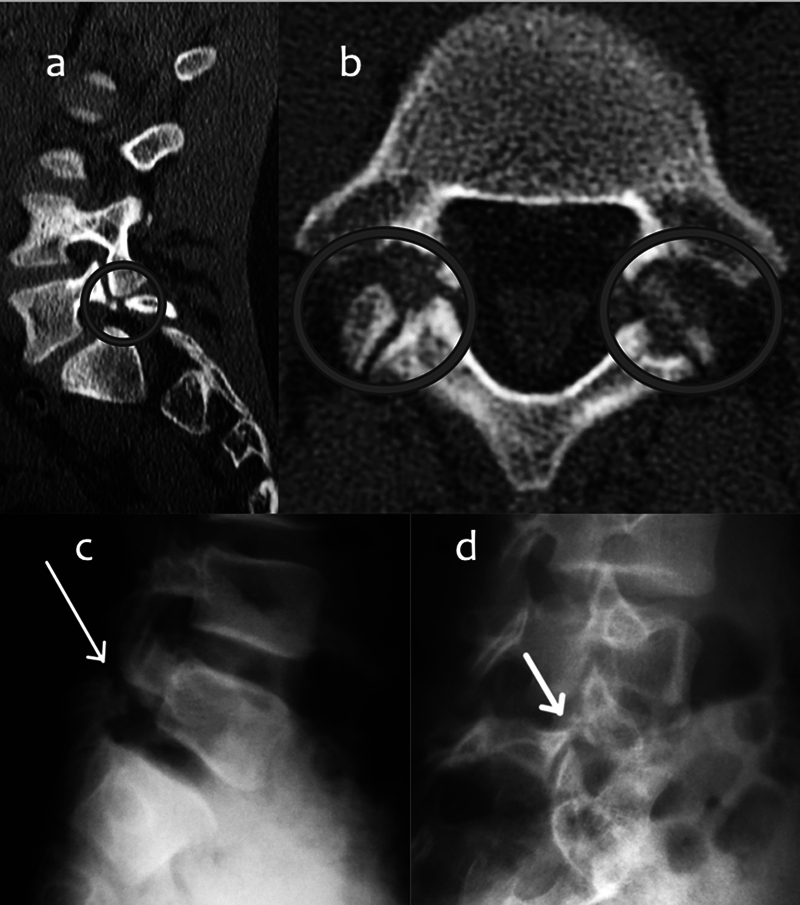
Examples of spondylolysis. ( **a** ) Computed tomography (CT), sagittal view. ( **b** ) Reverse gantry CT, axial view. **(c)** Simple collimated lateral radiograph. ( **d** ) Oblique radiograph showing the “Scotty dog” signal.

Spondylolysis is a lytic lesion in the posterior vertebral arch affecting mostly the pars interarticularis of L5; it can be unilateral or bilateral ( Fig. 1 ). 1 Since spondylolysis relates to the repetition of sporting gestures, especially under flexion-extension and trunk rotation movements, it is also considered a stress fracture. 2 Therefore, spondylolysis must be the leading initial diagnostic hypothesis in athletes with low back pain. 3 Moreover, there is an unknown but relevant percentage of asymptomatic athletes with this type of injury. 4 Spondylolisthesis consists of an anterior vertebral slippage to the distal segment. In the presence of pars lysis (spondylolysis), spondylolisthesis is a type 2a injury according to the modified Wiltse etiological classification. 5 The incidence of low back pain in athletes is high, reaching 86% per the literature; in addition, its association with spondylolysis in up to 60% of the cases demonstrates the need for an exact understanding of the natural history of this condition. 6

## Epidemiology

Nearly 93% of cases of spondylolysis are associated with sports practice. 7 Its incidence in the general population is 6%. About 75% of the subjects will progress to some degree of anterior slippage, i.e., spondylolisthesis. 8 Considering each modality separately, the impact sports most practiced in developed countries have the following incidences of spondylolysis: up to 44% in hockey players, 9 of which 15.9% also have listhesis, 10 40% in tennis players, 11 up to 40% in diving athletes, 9 20.69% in volleyball players, 12 and up to about 50% in cricket, rugby, and American football players. 13 14 15 16

## Clinical Diagnosis

Spondylolysis must be the leading diagnostic hypothesis in young athletes with low back pain until proven otherwise. 17 Any complaint lasting longer than two weeks warrants investigation. A detailed history, ruling out macrotrauma and previous injuries and including personal and family history, is essential. The most relevant information is a change in the training pattern (migration to a new sport modality, change in the amount/quality of exercise, loading increase to improve performance, etc.). 18 The next step must be a detailed physical examination. With the patient wearing swimming trunks (and preferably accompanied by someone else or a nurse), carry out the following: a) a static inspection, with an observation from all angles (front, back, and sides), to identify potential deformities (accentuated or diminished kyphosis or lordosis, scoliosis), asymmetries, and shoulder and pelvic tilts; and b) a dynamic inspection, observing gait and spine segment mobility during smooth flexion, extension, and rotation, completing the postural evaluation. Note that flexion and trunk extension can vary from +- 45 degrees; 19 also check hip movements. Proceed to palpation with the patient lying prone on a stretcher to identify any pain, muscle hypertonia, and anatomical points, including spinous processes, iliac wings, and beginning of sacroiliac joints (pain in this region indicates a positive Finger test). 20 Next, perform a neurological assessment; although spondylolysis barely affects the neurological function, this evaluation must always occur to determine the sensorimotor picture of the lower lumbar roots, namely: a) L4: medial dermatome of the leg and foot and tibialis anterior muscle; b) L5: lateral dermatome of the leg and dorsal foot and extensor hallucis longus muscle; c) S1: lateral dermatome of the foot and peroneus longus and brevis muscles - grade the motor strength in a scale from 0 (no strength) to 5 (normal strength). Also, test patellar (L4) and calcaneal (S1) tendon reflexes. 21 Special maneuvers include root irritation screening, such as the extended leg and Lasègue tests, and hip/sacroiliac maneuvers, such as the Patrick-Fabere, Gaeslen, and Finger tests. Jackson described trunk hyperextension with unipodal support as pathognomonic of spondylolysis; although contested in recent articles, this test remains the only one specific for this lesion. 22 23

## Imaging

Radiography reveals a radiolucent lesion in the pars interarticularis at the level investigated in collimated lateral and oblique views (the so-called “Scotty dog” sign) with a 97% accuracy for chronic spondylolysis (post-edema with an established fracture) ( Figs. 1c and 1d ). 24 Healing lesions present the typical sclerosis of bone callus in the anteroposterior view. 25 Lateral radiographs under maximal extension and flexion may indicate instabilities resulting from the increased slippage in the anterior direction (spondylolisthesis) greater than 4 mm or a tilt higher than 10 degrees between adjacent plateaus. 26

Computed tomography (CT) is still the best test to study complete or incomplete lesions with bone continuity for precise anatomical visualization. As such, CT is often requested for the preoperative planning of cases refractory to conservative treatment ( Fig. 1a ). The reverse gantry angle technique ( Fig. 1b ) provides a faithful image of the lesion and differentiates it from the joint facet (double facet sign). 27 CT reveals small, sclerotic, and hypertrophic reactions related to lesion evolution and differential diagnoses, such as osteoid osteoma. However, CT may be most valuable for postoperative consolidation follow-up. 28

The great advantage of magnetic resonance imaging (MRI) over CT is lesion detection at an early stage, i.e., identifying a medullary edema with no bone continuity loss in the pars. In the past, MRI accuracy was deemed insufficient for a safe spondylolysis diagnosis; this belief has been discredited due to the evolution of image acquisition techniques culminating in a specific classification. 29 Another obvious advantage of MRI is that it does not require radiation, unlike CT, avoiding its potentially undesirable effects. MRI is also the most effective way to point out foraminal stenoses, discopathies, and radicular anatomical changes; it may even detect neural tumors ( Fig. 4a ). 30

Although not part of our routine, bone scintigraphy can differentiate acute from chronic lesions, 31 like MRI.

**Fig. 2:**
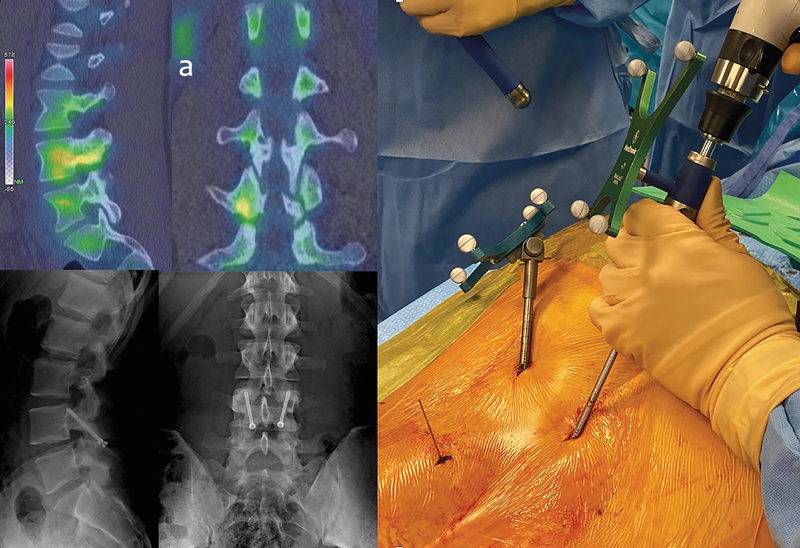
Percutaneous fixation under navigation. ( **a** ) Single-photon emission computed tomography-computed tomography (SPECT-CT) reveals a “hot lesion” in L3.

On the other hand, single-photon emission computed tomography-computed tomography (SPECT-CT) is more accurate than other tests 32 both for diagnosis and for anatomical location as it allows the differentiation of chronic (“cold”) and acute (“hot”) lesions. Due to SPECT-CT's high cost and radiation issues, we use it only when MRI does not clarify the diagnostic hypothesis ( Fig. 2a ).

## Classifications

MRI is the base for the most accepted spondylolysis classification. In this classification, MRI findings generate the following five groups: type 0 (no alterations), type 1 (edema with no cortical rupture), type 2 (bone irregularity demonstrating incomplete pars lesion), type 3 (acute lesion), and type 4 (chronic lesion). 29

At first treated as a local alteration, spondylolysis with spondylolisthesis was next evaluated with pelvic balance, then the entire spine, and finally, as a global alteration. This assessment allows the observation of compensatory knee flexion even in milder cases. 33

The traditional classifications for spondylolisthesis include the modified Wiltse classification, which is etiological. Type II refers to spondylolytic listhesis, and it consists of subtypes A (pars lysis, the most frequent spondylolytic listhesis in athletes), B (elongated pars), and C (traumatic injury). Meyerding 34 described another classification system based on the percentage of slippage (no slippage; up to 25% slippage; 25 to 50% slippage; 50 to 75% slippage; 75 to 100% slippage; and spondyloptosis, i.e., total slippage).

The Spinal Deformity Study Group (SDSG) classification for spondylolisthesis in L5-S1 (the most commonly affected level) considers the sacropelvic orientation.

Lateral radiographs evaluate the overall sagittal balance using the three following parameters: slippage degree, pelvic tilt, and spinopelvic alignment. These radiographs identify six injury types with progressive severity; the first three are low-grade lesions, the most common in athletes. This classification guides the surgical approach according to the need to restore the sagittal parameters. 35 In addition, an algorithm differentiates spondylolysis from acute nonspecific low back pain. The authors described the difficulty of finding lesion-specific clinical signs and their differentiation from nonspecific low back pain in imaging tests, such as radiography, which also does not have acceptable diagnostic accuracy. Therefore, diagnosis requires more complex, expensive tests, like CT and MRI.

## Risk Factors

The following intrinsic variables are associated with a greater risk of spondylolysis: male gender, occult spina bifida, 36 37 increased lordosis and pelvic tilt, 38 hamstring muscles shortening, and an imbalance of the anterior and posterior muscles that stabilize the trunk. 39 We also understand that the amount and quality of exercise are extrinsic determinant factors for this type of injury. 2 40

## Treatment

**Fig. 3:**
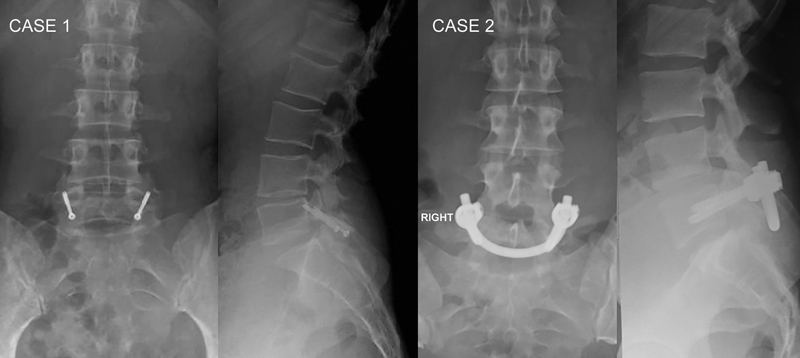
Postoperative radiographs. Case 1, modified Buck technique. Case 2, Smile technique.

Most cases respond well to conservative treatment, which consists of relative rest and rehabilitation with physical therapy. 41 We do not recommend braces in our routine for two reasons. First, braces result in disuse atrophy of the trunk-stabilizing paravertebral muscles; second, they do not respect the immobilization principle, i.e., blocking the range of motion in a proximal and a distal joint, since these orthoses do not act on the hips. 17 The plaster cast of Risser-Cotrel 42 provides hip blocking, but it is in total disuse due to the discomfort to the patient. Surgical treatment is reserved for cases with no improvement after at least six months of conservative treatment. Some authors recommended infiltration in the pars defect area to confirm the pain origin. 43 The first surgical procedure proposed for this type of lesion was non-instrumented in situ arthrodesis with a posterolateral graft. 44 Subsequently, scientific evidence indicated that the associated instrumentation significantly increased the fusion success rate, which was even higher with the inclusion of the three columns, i.e., 360-degree arthrodesis, either by a posterior route alone or combined with an anterior approach. We believe arthrodesis is not the optimal treatment due to medium-term loss of range of motion and adjacent degeneration, which are even more likely in athletes. 45 Here CA, are two techniques for direct pars repair with no arthrodesis and placing an autologous graft in the defect area ( Fig. 3 ). 46 47 48 49 50 This is our technique of choice in most cases, especially when the disc at the involved level presents no degeneration, a common finding in young athletes with a recent injury. This procedure has very satisfactory outcomes, with around 90% of the patients returning to the pre-injury sporting. Using intraoperative CT and neuronavigation allows a percutaneous approach; we believe that simplifying the procedure will change the protocol, shortening the time of conservative treatment and increasing surgical fixation indications ( Fig. 2 ). 17

**Fig. 4:**
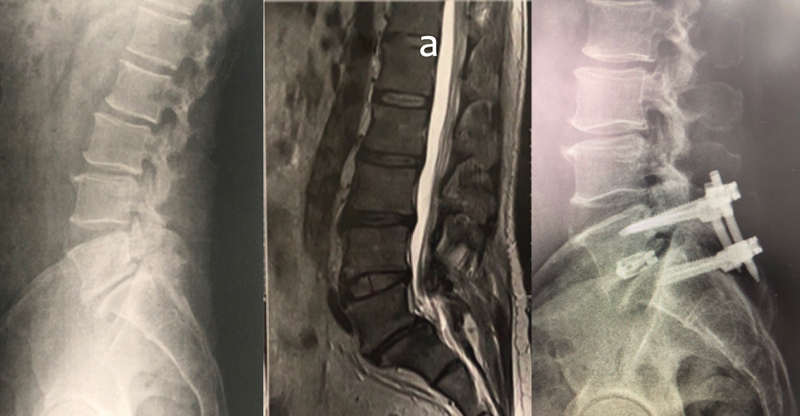
Arthrodesis with percutaneous screws and ENDOLIF. ( **a** ) Magnetic resonance imaging.

**Fig. 5:**
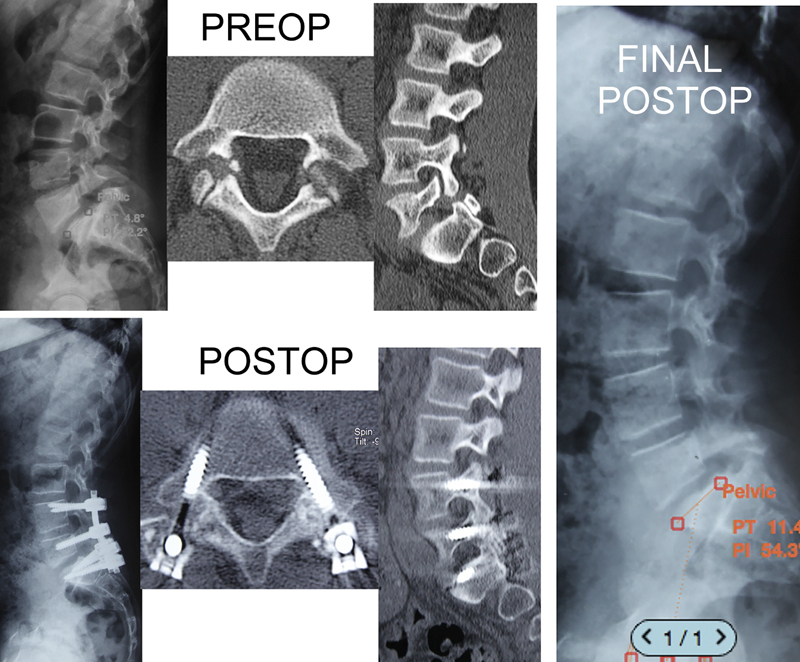
Slippage reduction with temporary fixation. We noted a significant improvement in sagittal parameters after synthesis material removal.

Moreover, it is possible to use an endoscopic technique for curettage and graft placement in the pars gap for percutaneous fixation, increasing the lesion consolidation rate and making the procedure minimally invasive. For cases of spondylolisthesis higher than grade I (more than 25% of slippage), advanced disc disease, or significant associated instability, we consider 360-degree arthrodesis with the placement of a spacer via the anterior approach (ALIF). Alternatively, we contemplate the posterior endoscopic approach using the Endoscopic Spinal Stabilization technique with EndoLIF® ( Fig. 4 ) complemented with percutaneous screws via the posterior approach. However, fusion has disadvantages, as already mentioned. Another option is a temporary reduction with pedicle screws with no arthrodesis (no posterolateral graft placement or facet opening), followed by synthesis material removal ( Fig. 5 ). The advantage of this technique is the lack of arthrodesis, but there is a risk of synthesis material breakage during consolidation of the pars interarticular failure.

## Complications

There are reports of immediate postoperative complications, including local infection, pain in the bone graft donor region, and synthesis material breakage, but at an extremely low rate (p = 0.011). 51

## Final Considerations

All low back pain cases for more than two weeks in young athletes must be considered a stress fracture until proven otherwise. Lumbar spondylolysis in athletes results from local overload during the repetitive effort of high-performance training. In young subjects, it may also occur with anterior slippage and spondylolytic spondylolisthesis. The prognosis is associated with early diagnosis and termination of impact activities. Lesion identification through imaging tests is paramount, and MRI seems to be the test of choice after negative radiographs. Doubtful cases may benefit from scintigraphy and SPECT-CT, when available. CT is reserved for chronic cases refractory to conservative treatment during surgical planning or follow-up to confirm consolidation. Conservative treatment is enough in the absolute majority of cases. However, surgical indications may be more frequent in professional athletes due to the long time away from sports.

**Table 1:** Modified Wiltse classification 5

TYPE	ETIOLOGY	PATHOGENESIS
I	Dysplastic	Congenital defect
II		Pars defect
IIa	Isthmic	Spondylolysis (stress fracture)
IIb		Pars stretching
IIc		Acute pars fracture
III	Degenerative	Facet subluxation
IV	Traumatic	Acute posterior column fracture
V	Pathological	Infection, tumor, etc.
VI	Postoperative	Postoperative instability

**Table 2:** Classification of spondylolysis according to magnetic resonance imaging (MRI) 29

GRADE	DESCRIPTION	MRI FINDING
**0**	Normal pars	Normal signal
		Intact cortical bone
**1**	Stress reaction	Medullary edema, intact cortical bone
**2**	Incomplete fracture	Medullary edema, incomplete cortical fracture
**3**	Acute complete fracture	Medullary edema, complete pars fracture
**4**	Established chronic defect	No medullary edema; complete pars fracture
