# Supplementary material for: Spondylolysis and Spondylolisthesis in Athletes
Source: Rev Bras Ortop (Sao Paulo). 2024 Mar 21;59(1):e10–6. doi: 10.1055/s-0043-1777435 (PMC10957283; doi:10.1055/s-0043-1777435)
Supplement: Related texts

# Research article: Espondilólise e espondilolistese em atletas

**Keywords:** atletas, espondilólise, espondilolistese, dor crônica, dor lombar, esporte

## Abstract

Este artigo é uma atualização do tema espondilólise e espondilolistese em atletas, do diagnóstico ao tratamento, baseando-se na experiência dos nossos serviços juntamente com uma revisão da literatura.

## Introdução

A espondilólise é uma lesão lítica no arco posterior vertebral, que na maioria dos casos acomete a *pars interarticularis* de L5, podendo se apresentar de forma uni ou bilateral ( Fig. 1 ). 1 Como está relacionada com mecanismos de repetição do gesto esportivo, em especial nos movimentos de flexo-extensão e rotação do tronco. Ela tambem é considerada uma fratura por estresse, 2 e, portanto, o atleta com lombalgia deve ter esse diagnóstico como a principal hipótese inicial. 3 Além disso há um percentual desconhecido, mas relevante de atletas assintomáticos com esse tipo de lesão. 4 A espondilolistese caracteriza-se por um escorregamento anterior da vértebra em relação ao segmento distal, e de acordo com a classificação etiológica de Wiltse modificada quando há lise da *pars* (espondilólise) associada se encaixa no tipo 2a. 5 A alta incidência de lombalgia em atletas pode chegar a 86%, de acordo com a literatura, e a associação deste quadro com espondilólise de até 60% demonstram a necessidade do exato entendimento da história natural da patologia. 6

**Fig. 1:** Exemplos de espondilólise. ( **a** ) corte sagital de tomografia, ( **b** ) corte axial de tomografia tipo pórtico reverso, ( **c** ) radiografia simples de perfil colimado e ( **d** ) radiografia oblíqua tipo “Scotty Dog”

## Epidemiologia

Cerca de 93% dos casos de espondilólise estão associados à pratica esportiva. 7 A sua incidência na população geral é de 6% e cerca de 75% dos casos evoluirão para algum grau de escorregamento anterior, ou seja, para uma espondilolistese. 8 Considerando cada modalidade separadamente, observamos que os esportes de impacto mais praticados em países desenvolvidos apresentam as seguintes incidências: até 44% em jogadores de *Hockey* , 9 sendo que 15,9% com listese associada, 10 40% em jogadores de tênis, 11 até 40% em mergulhadores de salto ornamental, 9 20,69% em jogadores de vôlei 12 e até cerca de 50% em jogadores de *Cricket, Rugby* e futebol americano. 13 14 15 16

## Diagnóstico Clínico

A espondilólise deve ser considerada como principal hipótese diagnóstica em jovem atleta com lombalgia até que se prove o contrário, 17 qualquer queixa com duração maior do que duas semanas deve ser investigada. Uma história detalhada, afastando macrotrauma, lesões prévias, antecedentes pessoais e familiares é fundamental, sendo que a informação mais relevante é a mudança do padrão de treinamento (migração para nova modalidade esportiva, mudança de quantidade/qualidade do exercício, aumento da carga para ganho de desempenho etc). 18 O exame físico detalhado deve ser o passo seguinte. Com o paciente usando sunga (e de preferência com a presença de acompanhante ou enfermagem), realizam-se as inspeções: a) estática, através da observação em todos os ângulos (frente, costas e laterais), identificando possíveis deformidades (cifose ou lordoses acentuadas ou diminuídas, escoliose), assimetrias, inclinações dos ombros ou bacia, e b) dinâmica, com observação da marcha e mobilidade dos segmentos da coluna através de movimentos suaves de flexão, extensão e rotações, completando a avaliação postural, sendo que a flexão e a extensão do tronco podem variar de +- 45 graus 19 e também dos quadris. Inicia-se então a palpação, com o paciente deitado em decúbito ventral em uma maca, identificando se há dor, hipertonia muscular e os pontos anatômicos como processos espinhosos, asas dos ilíacos e início das articulações sacro-ilíacas (caso dor na região, temos o teste de *Finger* positivo). 20 Segue-se com a avaliação neurológica, pouco afetada pela espondilólise, mas que deve sempre ser realizada, com a observação do quadro sensório-motor das raízes lombares baixas, a saber a) L4: dermátomo medial da perna e do pé e músculo tibial anterior, b) L5: dermátomo lateral da perna e dorsal do pé e extensor longo do hálux c) S1: dermátomo lateral do pé e músculos fibulares longo e curto, sendo a força motora na escala de 0 (sem força) a 5 (força normal). Ainda devem ser testados os reflexos dos tendões patelar (L4) e calcâneo (S1). 21 As manobras especiais devem incluir pesquisa de irritação radicular, como as da perna estendida e de *Lasègue* , e quadris/sacro- ilíacas, como os sinais de *Patrick-Fabere, Gaeslen e Finger* , mas em especial a hiperextensão do tronco com apoio unipodal, descrito por Jackson como patognomônico de espondilólise, que embora contestado em artigos mais recentes, ainda é aceito como o único específico para esta lesão. 22 23

## Diagnóstico Por Imagem

A radiografia apontará uma lesão por radiotransparência na *pars interarticularis* do nível investigado e observado nas incidências de perfil colimado e oblíquas (o tradicional “ *Scotty Dog* ”) com uma acurácia de 97% para casos de espondilólise crônica (pós-edema, quando a fratura está estabelecida ( Fig. 1c e 1d ). 24 Nas lesões em resolução ainda podemos observar uma esclerose típica de calo ósseo na incidência ântero-posterior. 25 Radiografias em perfil com extensão e flexão máximas podem indicar instabilidades através de aumento do escorregamento no sentido anterior (espondilolistese) maior de 4 mm ou inclinação maior de 10 graus entre os platôs adjacentes. 26

Para o estudo das lesões completas ou incompletas com solução de continuidade óssea a tomografia computadorizada (TC) ainda é o melhor exame para uma visualização anatômica precisa, sendo, portanto, mais utilizada para planejamento pré-operatório dos casos refratários ao tratamento conservador ( Fig. 1a ). A técnica em ângulo de pórtico reverso ( Fig. 1b ) entrega uma imagem fiel da lesão e ainda diferencia da faceta articular (sinal da dupla faceta). 27 Pequenas reações escleróticas e hipertróficas relacionadas à evolução da lesão e diagnósticos diferenciais como osteoma osteóide também são facilmente identificados através da TC, mas talvez a sua maior utilidade seja a avaliação da consolidação no pós-operatório. 28

A grande vantagem da ressonância magnética (RM) em relação à TC é a de detectar de forma precoce a lesão, ou seja, identificar um edema medular sem ainda estar presente a perda de continuidade óssea da *pars* . No passado acreditava-se que a sua acurácia não era suficiente para um diagnóstico seguro de um caso de espondilólise, fato que foi sendo desacreditado ao longo da evolução das técnicas de aquisição de imagem e que culminou em uma classificação especifica. 29 Outra vantagem evidente é não necessitar de radiação para a sua realização, ao contrário da TC, evitando, portanto, seus possíveis efeitos indesejáveis. A RM também é a mais efetiva para apontar estenoses foraminais, discopatias, alterações anatômicas radiculares e até detectar a presença de tumores neurais ( Fig. 4a ). 30

Apesar de não fazer parte da nossa rotina, a cintilografia óssea tem a capacidade de diferenciar a lesão aguda da crônica, 31 tal como na RM.

Já o S *ingle Photon Emission Computed Tomography* acoplado à Tomografia Computadorizada) SPECT-TC, tem acurácia superior aos outros exames, 32 tanto para o diagnóstico quanto para a sua localização anatômica, por possibilitar a diferenciação de lesões crônicas (“frias”) de agudas (“quentes”), porém devido ao seu alto custo e necessidade de radiação reservamos apenas para os casos nos quais a RM não tenha esclarecido a hipótese ( Fig. 2a ).

**Fig. 2:** Caso de fixação percutânea com navegação. ( **a** ) SPECT-CT evidenciando “lesão quente” em L3

## Classificações

A classificação para espondilólise mais aceita é baseada na RM. Os achados no exame podem ser divididos em cinco grupos, sendo o tipo 0 sem alterações, tipo 1 com edema sem ruptura da cortical, tipo 2 com irregularidade óssea demonstrando lesão incompleta da pars, tipo 3 representando a lesão aguda e tipo 4 a lesão crônica. 29

A principio tratada como uma alteração local, a espondilólise com espondilolistese passou a ser avaliada em conjunto com o balanço pélvico, em seguida com toda a coluna e por fim como uma alteração global. Desta forma é possível observar flexão compensatória dos joelhos até em casos mais leves. 33

As classificações tradicionais para espondilolistese são a de Wiltse modificada 5 que é etiológica, sendo que o tipo II trata da listese espondilolítica, podendo ainda ser dividida nos subtipos A (lise da *pars* , a mais comum em atletas), B ( *pars* alongada) e C (traumática), e a de *Meyerding* 34 que leva em conta a porcentagem de escorregamento (sem escorregamento, até 25%, de 25 a 50%, de 50 a 75%, de 75 a 100% e espondilopitose, o escorregamento total).

Também há a classificação do Spinal Deformity Study Group (SDSG) para espondilolistese L5-S1 (nível mais frequente) que leva em conta a orientação sacro-pélvica.

Na radiografia em perfil avalia-se o balanço sagital global através de 3 parâmetros: grau de escorregamento, incidência pélvica e alinhamento espino- pélvico. Foram identificados 6 tipos com gravidade progressiva sendo os 3 primeiros, de baixo grau, mais comuns em atletas. Esta classificação permite a orientação do tipo de abordagem cirúrgica de acordo com a necessidade de restauração dos parâmetros sagitais. 35 Um algoritmo ainda foi desenvolvido para diferenciar espondilólise de lombalgia inespecífica aguda. Os autores descrevem a dificuldade de achar sinais clínicos específicos para a lesão e como diferenciá-la de uma lombalgia inespecífica, levando em conta exames de imagem como a radiografia, que também não tem acurácia aceitável para o diagnóstico, restando apenas exames mais complexos e caros como a TC e a RM.

## Fatores de Risco

São reconhecidas como associadas a um maior risco de espondilólise as seguintes variáveis intrínsecas: sexo masculino, presença de espinha bífida oculta, 36 37 lordose e inclinação pélvica aumentadas, 38 encurtamento dos músculos isquiotibiais e desequilíbrio da musculatura anterior e posterior que estabiliza o tronco. 39 Entendemos também que a quantidade e a qualidade do exercício são fatores extrínsecos determinantes para a ocorrência deste tipo de lesão. 2 40

## Tratamento

A imensa maioria dos casos é resolvida com o tratamento conservador, que basicamente consiste em um repouso relativo e reabilitação com fisioterapia. 41 Não recomendamos em nossa rotina o uso de colete, pois além de causar uma atrofia muscular por desuso na musculatura paravertebral que estabiliza o tronco, o princípio da imobilização que consiste em bloquear amplitude de movimento em uma articulação proximal e uma distal não pode ser respeitado, pois, os quadris não são imobilizados com estes tipos de órteses. 17 Durante muito tempo foi preconizado o uso da imobilização gessada de *Risser-Cotrel* , 42 esta sim, proporciona o bloqueio dos quadris, porém em total desuso devido ao incômodo causado ao paciente. O tratamento cirúrgico fica reservado aos casos que não melhoram com no mínimo seis meses de tratamento conservador. Alguns autores recomendam uma infiltração na região do defeito da pars para confirmação da origem da dor. 43 O primeiro procedimento cirúrgico proposto para este tipo de lesão foi a artrodese *in situ* não instrumentada com colocação de enxerto póstero-lateral. 44 Posteriormente as evidências científicas indicaram que a instrumentação associada aumentou significativamente a taxa de sucesso da fusão, maior ainda com a inclusão das três colunas, ou seja, a artrodese 360 graus, seja por via posterior apenas ou combinada com a via anterior. A artrodese não deve ser considerada como forma ideal de tratamento na nossa visão, levando em conta a perda de amplitude movimento e a degeneração adjacente em médio prazo que será ainda mais provável no atleta. 45 Aqui, CA, existem duas técnicas de reparo direto da *pars* sem artrodese e colocação de enxerto autólogo na região da falha ( Fig. 3 ), 46 47 48 49 50 sendo esta a nossa técnica de escolha na maioria dos casos em especial se o disco do nível envolvido não estiver degenerado, o que é mais comum em atletas jovens com lesão recente. Os resultados deste procedimento são muito satisfatórios, cerca de 90% retorna ao mesmo nível esportivo de antes da lesão. Com a possibilidade do uso da TC intra-operatória associada a neuronavegação, que torna o procedimento percutâneo possível, acreditamos que, pela simplificação do procedimento, o protocolo será alterado, encurtando o tempo de tratamento conservador e consequentemente indicando com maior frequência a fixação cirúrgica ( Fig. 2 ). 17

**Fig. 3:** Radiografias pós-operatórias. Caso 1, técnica de Buck modificada. Caso 2, técnica de Smile.

Ainda é possível o uso da técnica endoscopia para curetagem e colocação de enxerto na falha da *pars* nos casos de fixação percutânea, aumentando a taxa de consolidação da lesão e tornando o procedimento minimamente invasivo. Para os casos de espondilolistese acima de grau I (mais de 25% de escorregamento), discopatia avançada ou instabilidade importante associada consideramos a artrodese 360 graus com colocação de espaçador por via anterior (ALIF) ou via endoscópica posterior pela tecnica de Endoscopic Spinal Stabilization with EndoLIF® ( Fig. 4 ) e complementação com parafusos percutâneos por via posterior, mas as desvantagens da fusão já foram citadas anteriormente. Uma outra opção é a redução com parafusos pediculares provisória sem a realização de artrodese (sem colocação de enxerto póstero-lateral ou cruentização das facetas) e em um segundo tempo realizar-se a retirada do material de síntese ( Fig. 5 ). A vantagem desta técnica é evitar a artrodese, porém há um risco de quebra do material de síntese durante o período de consolidação da falha da *pars interarticular.*

**Fig. 4:** Caso de artrodese com parafusos percutâneos e ENDOLIF. ( **a** ) ressonância magnética.

**Fig. 5:** Caso com redução do escorregamento com fixação temporária e após a retirada do material de síntese observada melhora importante dos parâmetros sagitais.

## Complicações

São relatadas complicações pós-operatórias imediatas como infecção local, dor na região doadora de enxerto ósseo quando realizado e quebra do material de síntese, todas em uma taxa extremamente baixa (p = 0,011). 51

## Considerações Finais

Toda dor lombar há mais de duas semanas em atleta jovem deve ser considerada como fratura por estresse até que se prove o contrário. A espondilólise lombar em atletas é causada por sobrecarga na região durante o esforço repetitivo do treinamento de alta performance. Nos mais jovens pode cursar ainda com escorregamento anterior, a espondilolistese espondilolítica. O prognóstico está ligado ao diagnóstico precoce e à suspensão das atividades de impacto. A identificação da lesão deve ser feita através de exames de imagem, e a RM parece ser o exame de escolha após radiografias negativas, e em casos de dúvida a cintilografia e o SPECT-TC quando disponíveis podem ajudar. A TC fica reservada para os casos crônicos refratários ao tratamento conservador, quando se planeja a cirurgia ou no acompanhamento para confirmação de consolidação. Na maioria absoluta dos casos o tratamento conservador costuma ser suficiente, embora devido ao longo tempo de afastamento do esporte os atletas profissionais podem passar a ter a cirurgia mais frequentemente indicada.

**Tabela 1:** Classificação de Wiltse modificada 5

TIPO	ETIOLOGIA	PATOGÊNESE
I	Displásica	Defeito congênito
II		Defeito da Pars
IIa	Ístmica	Espondilólise (fratura de estresse)
IIb		Alongamento da Pars
IIc		Fratura aguda da Pars
III	Degenerativa	Subluxação facetária
IV	Traumática	Fratura da coluna posterior aguda
V	Patológica	Infecção, tumor etc
VI	Pós-operatória	Instabilidade pós-operatória

**Tabela 2:** Classificação de espondilólise de acordo com a ressonância magnética 29

GRAU	DESCRIÇÃO	IMAGEM NA RM
**0**	Pars normal	Sinal normal
		Cortical intacta
**1**	Reação de estresse	Edema medular Cortical intacta
**2**	Fratura incomplete	Edema medular Fratura da cortical incompleta
**3**	Fratura aguda complete	Edema medular Fratura completa da Pars
**4**	Defeito crônico estabelecido	Sem edema medular Fratura completa da Pars
